# Trajectories of Neighborhood Cohesion in Childhood, and Psychotic and Depressive Symptoms at Age 13 and 18 Years

**DOI:** 10.1016/j.jaac.2017.04.003

**Published:** 2017-07

**Authors:** Francesca Solmi, Ian Colman, Murray Weeks, Glyn Lewis, James B. Kirkbride

**Affiliations:** a Division of Psychiatry, University College London, London, UK; b School of Epidemiology, Public Health and Preventive Medicine, University of Ottawa, Ottawa, Canada; c Directorate of Force Health Protection, Canadian Forces Health Services Group, Ottawa

**Keywords:** neighborhood social cohesion, psychotic experiences, depressive symptoms, Avon Longitudinal Study of Parents and Children (ALSPAC), cohort study

## Abstract

**Objective:**

Exposure to adverse social environments has been associated with psychotic and depressive symptoms in adolescence in cross-sectional studies, but the longitudinal relation is unclear. This study examined whether longitudinal trajectories of exposure to adverse social environments across childhood are associated with psychotic experiences and depressive symptoms in adolescence.

**Method:**

Data on participants from the Avon Longitudinal Study of Parents and Children (ALSPAC) were used to estimate longitudinal trajectories of childhood exposure to neighborhood cohesion (NC), discord (ND), and stress (NS) using latent class growth modeling. Logistic regression was used to examine the association between these trajectories and psychotic experiences and depressive symptoms at 13 and 18 years of age, adjusting for maternal psychopathology, participant sociodemographic and socioeconomic characteristics, and area-level deprivation.

**Results:**

A dose-response association was observed between higher NS and the odds of psychotic experiences at 13 years (medium NS, adjusted odds ratio [aOR] 1.25, 95% CI 1.05–1.49; high NS, aOR 1.77, 95% CI 1.30–2.40), whereas high levels of ND predicted psychotic experiences at 18 years (aOR 1.50, 95% CI 1.10–2.07). High levels of NC (aOR 1.43, 95% CI 1.02–1.71) and NS (aOR 1.55, 95% CI 1.07–2.26) were associated with increased odds of high depressive symptoms at 18 years in a dose-response fashion.

**Conclusion:**

Prolonged and more severe exposure to adverse social environments is associated with greater odds of developing psychotic and depressive symptoms in late adolescence.

Children and adolescents living in deprived neighborhoods appear to experience worse mental health outcomes than their peers from more affluent areas,^1^, ^2^ including more internalizing^3^, ^4^, ^5^, ^6^ and psychotic^7^ symptoms and greater mental health service use.^8^ These findings echo studies of adults that have shown that rates of clinical disorders such as schizophrenia^9^, ^10^, ^11^ and depression^12^, ^13^, ^14^, ^15^ are higher in more deprived environments. In adults, other aspects of the social environment (i.e., beyond deprivation) including low levels of social cohesion (i.e., the set of shared norms, trust, and networks within a community^16^) have been linked to schizophrenia^10^, ^17^, ^18^, ^19^ and depression risk.^15^, ^20^, ^21^, ^22^, ^23^ Studies also have shown that individuals with schizophrenia report greater cumulative lifetime exposure to social disadvantage (e.g., having lower levels of education and employment, experiencing greater social isolation, living in more deprived environments) compared with general population controls.^24^ However, very few studies have investigated whether neighborhood social cohesion is associated with mental health problems in adolescence. Although there is some evidence to support this possibility for depressive^3^, ^4^, ^6^ and psychotic^7^ symptoms, only 2 studies have used longitudinal data.^3^, ^7^ Furthermore, only one of these examined whether repeated exposure to neighborhood social cohesion affected later adolescent mental health outcomes,^3^ finding an association between persistent childhood exposure to low social cohesion and depressive symptoms in adolescence, which is consistent with what is observed for schizophrenia in adults.^24^ This has not been replicated for adolescent depressive symptoms and has not been tested for adolescent psychotic experiences.

Given this limited evidence, we investigated whether trajectories of neighborhood social cohesion were associated with psychotic experiences and depressive symptoms at 13 and 18 years of age in a large general population birth cohort. We hypothesized that more severe and prolonged exposure to low neighborhood social cohesion would be associated with greater psychotic and depressive symptoms in adolescence and that these effects would persist after adjustment for individual- and other neighborhood-level characteristics.

## Method

### Sample

The Avon Longitudinal Study of Parents and Children (ALSPAC) is a birth cohort study of children born to women in Avon (Bristol, UK) from April 1, 1991 through December 31, 1992. A total of 14,541 women (87% of those invited and 72% of those eligible) and 13,988 children who were alive at 1 year of age (99.5% of all livebirths, N = 14,062) were recruited into the study and followed from pregnancy onward through self-report questionnaires and clinic visits. All participating mothers gave informed written consent before recruitment. More details on recruitment, sample representativeness, and follow-up assessments have been published elsewhere.^25^

In this study, we included all children with complete data on psychotic experiences and depressive symptoms at 13 and 18 years of age who had exposure data available at 1 or more time points. For twins (n = 404; 2.62% of ALSPAC sample), only 1 (i.e., the first born) was included to minimize bias risk estimates owing to shared genetic and environmental exposures. The ALSPAC ethics and law committee and the local research ethics committees gave ethics approval for this study.

### Measurements

#### Exposures

The main exposure variables were maternally reported trajectories of neighborhood social cohesion (NC), discord (ND), and stress (NS). These were identified empirically, as reported by the mother by questionnaire during pregnancy, at 8 months postpartum, and when the child was approximately 2, 3, 5, 7, and 10 years old. At each wave, mothers were asked the same set of questions about their relationship with neighbors and the overall rating of the neighborhood. From 2 years of age onward, mothers were asked about the quality of the built and social environment (Table S1, available online). All questions were rated on Likert scales. We conducted exploratory factor analysis when the child was 2 years old (the earliest wave at which all neighborhood items were asked), which led to the identification of 3 neighborhood constructs (i.e., NC, ND, and NS) based on visual inspection of the scree plot (Figure S1, available online). Items loaded distinctively onto each factor, with very little cross-loading (Table S2, available online). This allowed us to create a sum score for each participant’s exposure to NC, ND, and NS at each wave, derived by summing participant item responses for all items that loaded above 0.4 on a given factor. In a sensitivity analysis, we obtained the same factor structure when the child was 10 years old, providing evidence of good reliability of the neighborhood constructs during childhood (data available from the authors). Using neighborhood data at each wave, we estimated longitudinal exposure trajectories for each construct using latent class growth modeling (LCGM). Full details are provided in the Statistical Analyses section.

#### Outcomes

Data on psychotic experiences and depressive symptoms were collected at 13 and 18 years of age during clinic assessments (psychotic experiences at the 2 time points, depressive symptoms at 13 years) and by postal questionnaires (depressive symptoms at 18 years). The Psychotic-Like Symptoms Interview (PLIKSI) is a semistructured interviewer-rated screening assessment composed of 12 questions derived from the Diagnostic Interview Schedule for Children Version IV^26^ and the Schedule for Clinical Assessment in Neuropsychiatry,^27^ which aims to detect delusions, hallucinations, and intrusive thoughts. From the interview total score, we derived a binary variable indicating whether symptoms were absent or suspected or definite. Participants whose psychotic experiences could have been attributed to sleep problems or fever were considered as not having a psychotic experience.^28^, ^29^ The Short Moods and Feelings Questionnaire (S-MFQ) is a 13-item questionnaire developed to screen for depressive symptoms in childhood and adolescence. Questions are recorded on a Likert scale (“not true,” “sometimes,” “true”) scored from 0 to 2. We used a cutoff score of at least 11 to denote the presence of depression.^30^, ^31^, ^32^ The tetrachoric correlation coefficients between these 2 outcome variables (PLIKSI and S-MFQ) at each time point were low (0.3 and 0.4 for 13 and 18 years, respectively).

#### Other Variables

We measured several potential confounders, including child gender and ethnicity (white versus non-white), any flu infection during pregnancy (yes versus no), maternal and paternal age, maternal social class (manual versus non-manual), highest academic qualification (vocational, secondary, or degree or higher), marital status (single, married, or divorced, separated, or widowed), and number of house moves reported in the 3 years before pregnancy (0, 1–2, 3–4, ≥5). We also measured childhood exposure to stressful life events (any versus none) from a battery of mother-reported answers on stressful life events when the child was 1 year, 2 years, 3 years 6 months, and 5, 6, 7, and 9 years old. In addition, we controlled for trajectories of maternal depression using data collected during pregnancy, at 8 weeks and 8 months postpartum, and when the child was approximately 2, 3, 5, 6, 8, and 11 years old. Maternal depression was rated using the 10-item Edinburgh Postnatal Depression Scale,^33^ which estimates depressive symptoms in the previous week, rated on a 4-point Likert scale (“never” to “yes, most of the time/quite often”). Total scores were dichotomized (no symptoms versus probable depression) using a validated cutoff of 13.^33^, ^34^, ^35^ Trajectories were estimated using LCGM (see below for details of the modeling procedure). A 2-class latent trajectory solution (never had depression versus always had depression) provided the best fit (Figure S2, available online), which we subsequently used to control for maternal depression. We included a measurement of neighborhood deprivation during pregnancy, measured with quintiles of the Townsend Deprivation Index.^36^ The Townsend index is an indicator of material deprivation at the neighborhood level derived from 4 census variables (proportions of households without a car, overcrowded houses, households not occupied by owner, and persons unemployed). In our data, the Townsend index was positively correlated with measurements of ND and NS and negatively correlated with measurements of NC (Table S3, available online).

The ALSPAC website contains details of all the available data in a fully searchable data dictionary (http://www.bris.ac.uk/alspac/researchers/data-access/data-dictionary/).

### Statistical Analyses

#### Latent Class Growth Modeling

We classified participants into distinct exposure trajectories for childhood exposure to each neighborhood construct (NC, NS, and ND), based on exposure scores at each available wave, using LCGM (user-written Stata command traj).^37^ To identify the optimum number of trajectories, we jointly inspected the Bayesian information criterion parameter and posterior probabilities distributions (values > 70% indicating good model fit) and assigned participants to their most likely trajectory. To assess trajectory fit, we assessed the difference in Bayesian information criterion scores between 2 models, as suggested in the literature.^38^ The difference in Bayesian information criterion scores was multiplied by 2, with values from 0 to 2 indicating low evidence of model improvement, values from 4 to 6 indicating moderate evidence, values from 6 to 10 indicating strong evidence, and values higher than 10 indicating very strong evidence.^38^

#### Neighborhood Characteristics, Psychotic Experiences, and Depressive Symptoms

We ran 3 logistic regression models for each exposure and outcome combination: a univariable model (model I), a multivariable model adjusted for all confounders (model II), and a final model with additional mutual adjustment for the other neighborhood constructs (model III).

#### Missing Data

We included participants with complete outcome data at 13 years of age (psychotic experiences, n = 6,455; S-MFQ, n = 4,426) and 18 years of age (psychotic experiences, n = 6,378; S-MFQ, n = 3,231) who also had sufficient exposure data (from ≥1 time point) to assign them to a trajectory for each exposure (NC, NS, and ND). Missing covariate data were assumed to be missing at random and therefore imputed using multiple imputations with chained equations (MICE) and Stata command “ice”^39^ imputing 100 datasets and using linear, logistic, ordinal, and multinomial regression models according to the nature of the variable. In the imputation model, we included all variables used in the regression models and 2 other variables associated with missingness or other covariates (household income at 33 months and IQ at 8 years of age using the Wechsler Intelligence Scale for Children, Third Edition).^40^ All analyses were run using Stata 13.^41^

## Results

### Latent Class Growth Modeling

**Figure 1:**
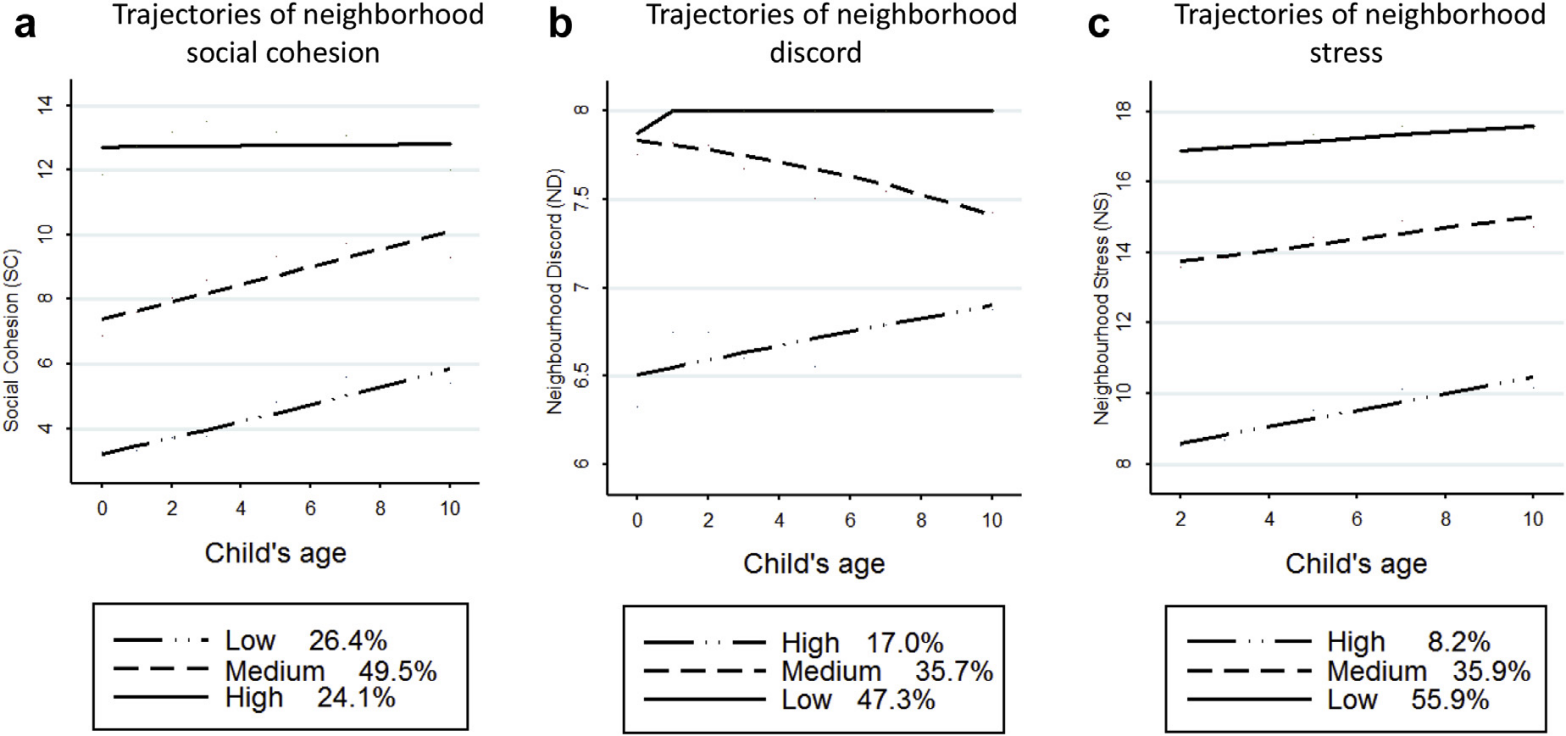
Trajectories of neighborhood social cohesion.

For each exposure, LCGM suggested that a 3-class solution (low, medium, and high NS, ND, and NC; Figure 1) provided a better fit to the data (Table S4, available online).

### Missing Data

The proportion of missing data for the covariates included in the analyses ranged from 0% to 45.3%, depending on the given outcome and covariate of interest (Table S5, available online). Participants with missing outcome data at 13 and 18 years of age were more likely to live in more deprived neighborhoods, with low levels of NC and high levels of ND; they also were more likely to be boys from a non-white ethnic background. Their mothers were more likely to be younger, not married, in non-manual occupations, less educated, to have experienced maternal depression, and to have moved house more often before pregnancy (Table S6, available online).

### Sample Characteristics

The sample size for each outcome at 13 and 18 years varied from 3,231 to 6,455, depending on the number of participants who completed each outcome assessment (Table 1). Most children included in the analyses were girls, of white ethnicity, and without experiences of stressful life events. Most mothers were married, had obtained at least a general certificate of secondary education qualification, had non-manual occupations, had moved houses fewer than 4 times before pregnancy, did not have flu during pregnancy, had never suffered from depression, and lived in neighborhoods in the least-deprived quintile (Tables S7, S8, available online). Most children lived in neighborhoods characterized by medium or high NC and low ND and NS (Table 1).

**Table 1:** Sample Distribution of Exposure Variables Across Outcome Levels

Exposure Variables	Psychotic Experiences at 13 y			Psychotic Experiences at 18 y		
	None, n (%)	Suspected or Definite, n (%)	*p* (χ^2^)	None, n (%)	Suspected or Definite, n (%)	*p* (χ^2^)
Total	5,719 (88.6)	736 (11.4)		4,084 (92.3)	342 (7.7)	
Neighborhood cohesion			.49			.07
High	1,448 (25.3)	176 (23.9)		1,071 (26.2)	81 (23.7)	
Medium	2,961 (51.8)	398 (54.1)		2,128 (52.1)	169 (49.4)	
Low	1,310 (22.9)	162 (22.0)		885 (21.7)	92 (26.9)	
Neighborhood discord			.44			<.0001
High	709 (12.4)	101 (13.7)		462 (11.3)	66 (19.3)	
Medium	1,471 (25.7)	196 (26.6)		1,054 (25.8)	91 (26.6)	
Low	3,539 (61.9)	439 (59.7)		2,568 (62.8)	185 (54.1)	
Neighborhood stress			<.0001			<.0001
High	343 (6.0)	77 (10.4)		228 (5.6)	39 (11.4)	
Medium	2,040 (36.7)	292 (39.7)		1,432 (35.1)	137 (40.1)	
Low	3,336 (58.3)	367 (49.9)		2,424 (59.4)	166 (48.5)	

Exposure Variables	Depressive Symptoms at 13 y			Depressive Symptoms at 18 y		
	No, n (%)	Yes, n (%)	*p* (χ^2^)	No, n (%)	Yes, n (%)	*p* (χ^2^)
Total	5,936 (93.1)	442 (6.9)		2,537 (78.5)	694 (21.5)	
Neighborhood cohesion			.1			.003
High	1,486 (25.0)	120 (27.2)		677 (26.7)	153 (22.1)	
Medium	3,105 (52.3)	208 (47.1)		1,306 (51.5)	352 (50.7)	
Low	1,345 (22.7)	114 (25.7)		554 (21.8)	189 (27.2)	
Neighborhood discord			.1			.03
High	739 (12.5)	67 (15.2)		245 (9.7)	91 (13.1)	
Medium	1,517 (25.6)	122 (27.6)		655 (25.8)	180 (25.9)	
Low	3,680 (61.9)	253 (57.2)		1,637 (64.5)	423 (61.0)	
Neighborhood stress			.004			<.0001
High	370 (6.2)	42 (9.5)		128 (5.1)	53 (8.5)	
Medium	2,128 (35.9)	173 (39.1)		842 (33.2)	225 (38.0)	
Low	3,438 (57.9)	227 (51.4)		1,567 (61.8)	314 (53.5)	

### Psychotic Experiences

At 13 and 18 years of age, 736 (11.4%) and 342 (7.7%) children had suspected or definite psychotic experiences (Table 1). At 13 years, more of these children had been exposed to persistently high NS in childhood (Table 1), whereas at 18 years more participants reporting psychotic experiences had lived in neighborhoods characterized by high levels of NS and ND and low NC during childhood compared with those not reporting psychotic experiences at these ages.

After multivariable logistic regression, there was evidence of a dose-response association between greater NS and increased risk of psychotic experiences at 13 years (model III; Table 2), but no evidence that NC and ND were associated with psychotic symptoms at that age. At 18 years, a crude association between NS and psychotic experiences did not persist after complete multivariable adjustment (model III); however, we observed that high exposure to ND was associated with a risk of psychotic experiences. As at 13 years, NC was not associated with psychotic experiences at 18 years after multivariable adjustment (Table 2).

**Table 2:** Logistic Regression Model Results (Odds Ratio [OR], 95% CI) for the Association Between Neighborhood Trajectories and Psychotic and Depressive Symptoms at 13 and 18 Years of Age (100 Imputations)^a^

Variables	Psychotic Experiences at 13 y			Psychotic Experiences at 18 y		
	Model I, OR (95% CI) (n = 6,455)	Model II, OR (95% CI) (n = 6,455)	Model III, OR (95% CI) (n = 6,455)	Model I, OR (95% CI), (n = 4,426)	Model II, OR (95% CI) (n = 4,426)	Model III, OR (95% CI) (n = 4,426)
Neighborhood cohesion						
High	reference	reference	reference	reference	reference	reference
Medium	1.11 (0.92–1.34)	1.04 (0.86–1.26)	1.02 (0.84–1.24)	1.05 (0.80–1.38)	0.91 (0.68–1.20)	0.92 (0.69–1.23)
Low	1.02 (0.81–1.28)	0.92 (0.73–1.17)	0.89 (0.70–1.13)	1.37 (1.01–1.88)‡	1.04 (0.75–1.45)	1.08 (0.78–1.51)
Neighborhood discord						
Low	reference	reference	reference	reference	reference	reference
Medium	1.07 (0.90–1.28)	0.99 (0.83–1.19)	0.95 (0.79–1.15)	1.20 (0.92–1.56)	1.09 (0.83–1.42)	1.07 (0.82–1.40)
High	1.15 (0.91–1.45)	0.98 (0.77–1.24)	0.90 (0.71–1.15)	1.98 (1.47–2.67)‡	1.57 (1.15–2.22)‡	1.50 (1.10–2.07)‡
Neighborhood stress						
Low	reference	reference	reference	reference	reference	reference
Medium	1.30 (1.10–1.53)‡	1.24 (1.04–1.48)‡	1.25 (1.05–1.49)‡	1.40 (1.10–1.77)‡	1.20 (0.93–1.54)	1.17 (0.91–1.51)
High	2.04 (1.56–2.67)‡	1.72 (1.27–2.34)‡	1.77 (1.30–2.41)‡	2.50 (1.72–3.63)‡	1.60 (1.04–2.45)‡	1.47 (0.95–2.27)

Variables	Depressive Symptoms at 13 y			Depressive Symptoms at 18 y		
	Model I, OR (95% CI) (n = 6,378)	Model II, OR (95% CI) (n = 6,378)	Model III, OR (95% CI) (n = 6,378)	Model I, OR (95% CI) (n = 3,231)	Model II, OR (95% CI) (n = 3,231)	Model III, OR (95% CI) (n = 3,231)
Neighborhood cohesion						
High	reference	reference	reference	reference	reference	reference
Medium	0.83 (0.66–1.04)	0.80 (0.63–1.01)	0.86 (0.63–1.02)	1.19 (0.97–1.47)	1.15 (0.92–1.42)	1.15 (0.92–1.43)
Low	1.05 (0.80–1.37)	1.01 (0.76–1.33)	1.01 (0.76–1.34)	1.51 (1.19–1.92)‡	1.33 (1.03–1.71)‡	1.43 (1.02–1.71)‡
Neighborhood discord						
Low	reference	reference	reference	reference	reference	reference
Medium	1.17 (0.93–1.46)	1.11 (0.88–1.39)	1.08 (0.88–1.36)	1.06 (0.87–1.29)	1.02 (0.83–1.25)	1.00 (0.82–1.24)
High	1.32 (1.00–1.75)†	1.18 (0.88–1.58)	1.12 (0.83–1.51)	1.44 (1.10–1.87)‡	1.24 (0.93–1.63)†	1.18 (0.88–1.56)
Neighborhood stress						
Low	reference	reference	reference	reference	reference	reference
Medium	1.23 (1.00–1.51)†	1.20 (0.96–1.49)	1.19 (0.95–1.48)	1.32 (1.11–1.58)‡	1.26 (1.04–1.52)‡	1.24 (1.02–1.50)‡
High	1.72 (1.22–2.43)‡	1.48 (1.00–2.19)†	1.44 (0.97–2.14)	1.95 (1.40–2.71)‡	1.63 (1.13–2.35)‡	1.55 (1.07–2.26)‡

Note: Model I = crude model; model II = maternal and paternal age; maternal education, marital status, social class, depression, number of house moves, flu during pregnancy; child’s ethnicity, gender, stressful life events, quintiles of area deprivation in pregnancy; model III = model II mutually adjusted for all exposure variables.

a Number refers to children who have complete outcome data, exposure measurements at at least 1 time point, and firstborn in case of twin births; hence, it differs for each exposure and outcome combination and from the overall number reported in Table 1.

† .1 > *p* > .05;

‡ *p* ≤ .05.

### Depression

At 13 years, 442 children (6.9%) had high depressive symptoms, which increased to 694 (21.5%) participants at 18 years (Table 1). At 13 years, a larger proportion of children reporting high depressive symptoms had been living in neighborhoods characterized by high NS during childhood. A similar pattern was found at 18 years, when participants with depressive symptoms also were more likely to have lived in neighborhoods with high maternal-reported ND and low NC (Table 1).

At 13 years, we observed a crude association between higher levels of ND and NS and greater odds of reporting depressive symptoms, but these did not persist after full multivariable adjustment (model III; Table 2). At 18 years, low NC and higher NS were significantly associated with greater odds of depressive symptoms after mutual adjustment for all exposure variables (model III; Table 2), with some evidence of a dose-response association observed for NS.

## Discussion

In a large population-based birth cohort in England followed from birth until 18 years old, we found that children who were persistently exposed to greater neighborhood social adversity had higher odds of reporting psychotic experiences and depressive symptoms at 13 and 18 years after controlling for a number of confounders including maternal deprivation, family socioeconomic status, and area-level deprivation. Importantly, the specific type of neighborhood adversity associated with adolescent mental health varied; at 13 years, NS emerged as the strongest predictor of psychotic experiences and depressive symptoms, whereas at 18 years, lower NC and higher NS were more strongly associated with depressive symptoms than with psychotic experiences, which were predicted by greater ND.

Although the literature on this subject is sparse, our findings support limited available evidence that suggests that lower NC and NS are associated with greater depressive^3^, ^4^, ^5^, ^6^ and psychotic^7^ symptoms in children and adolescents. Only 1 previous study used a similar trajectory-based approach to ours (in relation to adolescent depressive symptoms only).^3^ They observed a U-shaped relation, such that childhood exposure to high and low NC was associated with increased internalizing problems. Although lower childhood NC also predicted greater depressive symptomatology in adolescence in our study, we did not find evidence of this U-shaped relation. Several factors could explain this, including different measurements used to ascertain adolescent psychiatric outcomes and maternal reporting of the neighborhood environment. For example, in our study, neighborhood questions referred primarily to the mother’s relationship with her neighbors; in the study by Kingsbury *et al.*,^3^ questions captured broader neighborhood dynamics, which might have tapped into other aspects of social capital (e.g., informal social control) not studied here.

To our knowledge, only 1 previous study has investigated the longitudinal association between childhood neighborhood environments and psychotic symptoms in adolescence in a nonclinical population.^7^ That study found an association between lower social cohesion and greater psychotic symptoms. We believe our findings are broadly consistent with this observation; although our measurement of NC was not associated with psychotic experiences directly, we found that childhood exposure to other closely related aspects of the neighborhood social environment (i.e., NS and ND) were predictive of adolescent psychotic experiences, with some evidence of a dose-response association.

Most other epidemiologic studies of neighborhood effects on child and adolescent mental health have used cross-sectional^1^, ^2^, ^4^, ^5^, ^6^ or case-control^8^ designs, limiting causal inference. This problem persists in regard to similar studies of adulthood psychiatric outcomes.^10^, ^13^, ^15^, ^17^, ^18^, ^19^ In contrast, our prospective longitudinal design enabled us to gain traction on the temporal relation between exposure and outcome, thus minimizing the likelihood that our results were due to reverse causation or recall bias.

Interestingly, there was some evidence that the observed associations were generally stronger and more consistent at 18 than at 13 years of age, particularly for depressive symptoms. Psychological symptoms at 13 years might have less specificity toward clinical phenotypes than those that occur closer to adulthood.^42^ Alternatively, families could play an important role in buffering the negative effects of neighborhood social adversities at younger ages, but this effect diminishes throughout adolescence, as the child gains greater independence and forms social networks extending beyond the immediate family nucleus. More research on this issue is required in longitudinal research of adolescent mental health.

Our data and those of other longitudinal studies in children and adolescents^3^, ^7^ suggest that neighborhood social adversity could operate to shift the entire population distribution of psychiatric symptomatology toward the phenotypic expression of clinical disorder.^43^ If true, then it is worth considering whether the specific patterns of association we observed—between NC and adolescent depressive symptoms, on the one hand, and ND and psychotic experiences, on the other (with some evidence that NS was related to the 2 outcomes)—merit further investigation. Although we could not exclude the possibility that these differences arose by chance, particularly for trajectories of ND, which were based on 2 questionnaire items, distinct etiologic differences between emergent psychotic and affective symptomatologies also might be apparent.^44^ For example, it has been suggested that stressful neighborhood environments could increase the risk of depression directly (e.g., through daily exposure to environmental stressors) and indirectly (e.g., by hampering the formation of social relationships within the neighborhood).^45^ Our finding that childhood exposure to low NC and high NS predicted greater depressive symptomatology at 18 years is in line with this hypothesis. Conversely, the association between high ND and psychotic experiences is in line with literature positing that exposure to hostile environments^46^ and trauma^47^ could act to increase psychosis risk.

Our study has a number of strengths. We used rich data from a large prospective birth cohort, including repeated measurements of childhood neighborhood environments, and several potential confounders at the family and area levels. Outcomes were measured with valid and reliable questionnaires (S-MFQ) and semistructured interviews (PLIKSI) that were previously used and validated.^28^, ^30^, ^48^ By using multiple imputation to handle missing values, we could increase our sample size and statistical power, thus limiting the potential for biases arising from selective attrition in the cohort.

Despite these strengths, a number of limitations merit discussion. First, our repeated measurements of the neighborhood social environment were ascertained by maternal self-report. If mothers of children who reported adolescent mental health problems also were more likely to report negative neighborhood characteristics, perhaps as a consequence of their own underlying maternal mental health, then we could have increased the risk of attributing genetic origins of adolescent mental health problems to social determinants. To mitigate this, we controlled for trajectories of maternal depression, which did not substantially attenuate our findings. Previous studies also have relied on self-reported measurements of the social environment.^3^, ^4^, ^6^, ^7^ Such perceptions also might be more relevant exposures than objectively rated measurements^49^; in our study, material deprivation did not confound associations with the perceived social environment. We could not determine whether maternal perceptions of the childhood neighborhood social environment accorded with the child’s perceptions, although this might be an important route of transmission through which deleterious or beneficial neighborhood environments affect subsequent adolescent mental health.

Second, we did not have mother-reported measurements of neighborhood quality when the child was 10 to 18 years old, a period when exposure to neighborhood adverse environments could have a stronger effect on children as they transition to independence. Our measurements of social cohesion have not been previously validated. However, they map onto constructs of social cohesion and trust and neighborhood safety, which have been previously used in the literature with similar results.^6^, ^8^

Third, although we used a validated cutoff for the S-MFQ to define depressive symptoms, we grouped together suspected and definite psychotic experiences in relation to PLIKSI scores, which could explain the presence of some stronger associations in relation to depression. Although this was based on considerations of statistical power and use of these variables in the previous literature,^28^, ^29^ the prevalence of depressive symptoms in this sample was higher than expected in a population of this age.^50^

Fourth, our LCGM models allowed us to identify distinct trajectories of membership to perceived neighborhood experiences, as reported by the mother, during childhood. This showed that trajectories were relatively stable over time, implying that early life exposure to a given level of cohesion, stress, or discord predicts ongoing exposure throughout childhood. Nonetheless, our approach had some limitations. We used a sum-score method to estimate neighborhood factors at each time point. Although sensitivity analysis showed that our derived factors were consistent at 2 and 10 years of age, we did not estimate exploratory factor analyses at every time point, which might have allowed more precise estimation of continuous factor scores. That said, cross-loadings were very weak, and a sum-score method should have led to reliable exposure estimation. We used a 3-stage modeling approach to estimate NC at each time point and then ran LCGM and conduct logistic regression with multiple imputations on the relation between neighborhood trajectories and depressive and psychotic symptoms in adolescence. Other approaches, including multilevel modeling, might have allowed for concurrent estimation of these effects, but we sought to ensure our results were comparable with the only other study on this topic to date.^3^

Fifth, there was notable attrition from our sample in late adolescence, resulting in smaller samples compared with early adolescence. Nonetheless, we detected several associations between our exposures and outcomes at 18 years, limiting the effect of type II error. We also used multiple imputations to account for missing exposure and covariate data within our available samples, strengthening the internal validity of our results. Whether our results are generalizable to the wider population would depend on patterns of attrition from our sample. Recent findings have indicated that children who are “at risk” for schizophrenia (i.e., based on genetic predisposition) are more likely to be lost to follow-up, suggesting that, if an association between neighborhood adversity and psychosis exists, we might have underestimated its magnitude.^51^

In conclusion, our study extends previous longitudinal research to suggest that childhood neighborhood social environments can alter the risk of developing psychotic and depressive symptoms in early and late adolescence. Our results support the possibility that interventions aimed at increasing aspects of the childhood neighborhood social environment, including improving cohesion and decreasing stress and discord, could lead to improved mental health and well-being in adolescence, a primary time for the emergence of mental health problems.
