# Corrigendum

**DOI:** 10.1016/j.jaac.2018.04.006

**Published:** 2018-06

In the research article “Trajectories of Neighborhood Cohesion in Childhood, and Psychotic and Depressive Symptoms at Age 13 and 18 Years” by Francesca Solmi, Ian Colman, Murray Weeks, Glyn Lewis, and James B. Kirkbride, published in the July 2017 issue of the *Journal of the American Academy of Child and Adolescent Psychiatry* (2017;56:570-577), an error appeared in the Results section of the Abstract. The sentence as published reads: “**High levels of NC** (aOR 1.43, 95% CI 1.02–1.71) **and NS** (aOR 1.55, 95% CI 1.07–2.26) were associated with increased odds of high depressive symptoms at 18 years in a dose-response fashion.” The sentence should instead read: “**Low levels of NC** (aOR 1.43, 95% CI 1.02–1.71) **and high levels of NS** (aOR 1.55, 95% CI 1.07–2.26) were associated with increased odds of high depressive symptoms at 18 years in a dose-response fashion.”

