# Supplementary material for: Trajectories of Neighborhood Cohesion in Childhood, and Psychotic and Depressive Symptoms at Age 13 and 18 Years
Source: J Am Acad Child Adolesc Psychiatry. 2017 Jul;56(7):570–7. doi: 10.1016/j.jaac.2017.04.003 (PMC5493518; doi:10.1016/j.jaac.2017.04.003)
Supplement: Tables S1-S8 and Figures S1 and S2 [file mmc1.docx]

**SUPPLEMENTARY MATERIAL**

**Table S1: List of Neighborhood Questions Asked in the Avon Longitudinal Study of Parents and Children (ALSPAC)**

| **Question ID** | **Question** | **Time Point Asked** |
| --- | --- | --- |
| **Q1** | Do the other people in your neighborhood visit your home? | **A, F, G, H, K, M, Q** |
| **Q2** | Do the other people in your neighborhood argue with you? | **A, F, G, H, K, M, Q** |
| **Q3** | Do the other people in your neighborhood look after your children? | **A, F, G, H, K, M, Q** |
| **Q4** | Do the other people in your neighborhood keep to themselves? | **A, F, G, H, K, M, Q** |
| **Q5** | Do you visit the homes of your neighbors? | **A, F, G, H, K, M, Q** |
| **Q6** | Do you argue with your neighbors? | **A, F, G, H, K, M, Q** |
| **Q7** | Do you look after your neighbors’ children? | **A, F, G, H, K, M, Q** |
| **Q8** | Do you keep to yourself? | **A, F, G, H, K, M, Q** |
| **Q9** | What is your opinion of your neighborhood? | **A, F, G, H, K, M, Q** |
| **Q10** | How much of a problem is noise from other people’s homes? | **G, H, K, M, Q** |
| **Q11** | How much of a problem is noise from outside in the street? | **G, H, K, M, Q** |
| **Q12** | How much of a problem is rubbish or litter dumped in the neighborhood? | **G, H, K, M, Q** |
| **Q13** | How much of a problem is dog dirt on pavements/walkways? | **G, H, K, M, Q** |
| **Q14** | How much of a problem is worrying about vandalism? | **G, H, K, M, Q** |
| **Q15** | How much of a problem is worrying about burglaries? | **G, H, K, M, Q** |
| **Q16** | How much of a problem is worrying about mugging or attacks? | **G, H, K, M, Q** |
| **Q17** | How much of a problem is disturbance from teenagers or youths? | **G, H, K, M, Q** |

**Age at time point:**

- **A =** Pregnancy
- **F =** 8 months postpartum
- **G =** 21 months (~2 years)
- **H =** 33 months (~3 years)
- **K =** 61 months (~5 years)
- **M =** 85 months (~7 years)
- **Q =** 122 months (~10 years)

**Figure S1: Scree plot.**

**
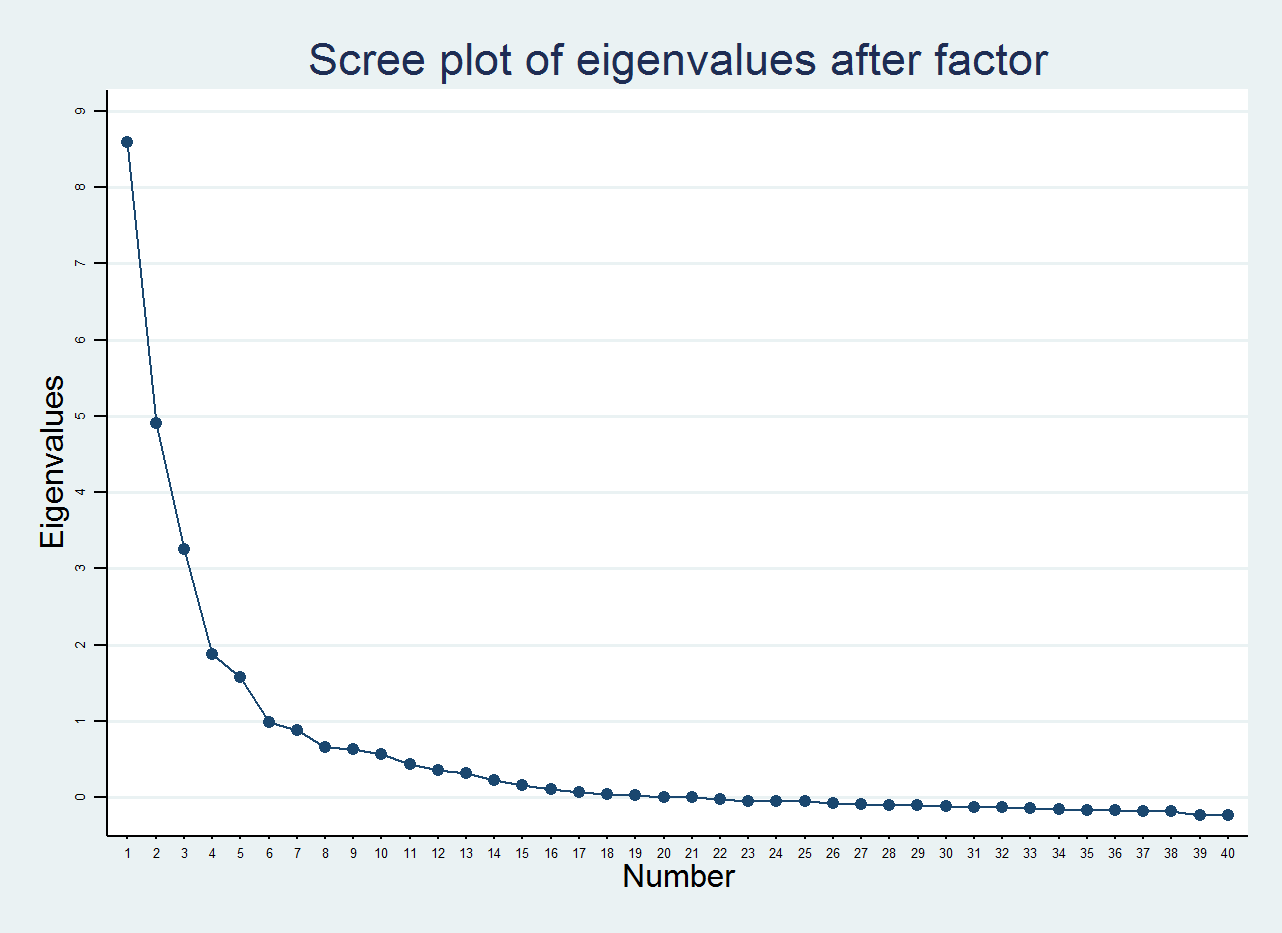
**

**Figure S2: Trajectories of maternal depression from birth to age 10 years of the child.**


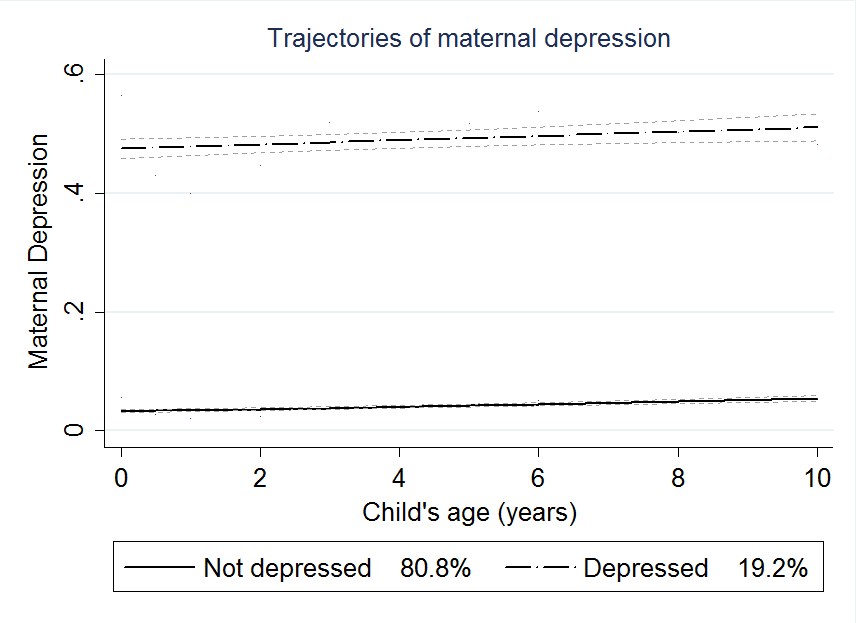


**Table S2: Exploratory Factor Analysis (EFA) for Questions at Time Point G (i.e., Child’s Age = 21 Months)**

| **Question ID** | **FACTOR 1**  **Neighborhood Social Cohesion and Trust**  **(factor loading)** | **FACTOR 2**  **Neighborhood**  **Discord**  **(factor loading)** | **FACTOR 3**  **Neighborhood**  **Stress/Quality**  **(factor loading)** |
| --- | --- | --- | --- |
| **Q1** | **0.83** | -0.02 | 0.00 |
| **Q2** | -0.06 | **0.95** | 0.00 |
| **Q3** | **0.72** | -0.13 | 0.00 |
| **Q4** | **0.75** | 0.05 | -0.01 |
| **Q5** | **0.85** | -0.00 | 0.01 |
| **Q6** | -0.06 | **0.96** | -0.00 |
| **Q7** | **0.75** | -0.14 | -0.03 |
| **Q8** | **0.77** | 0.12 | -0.01 |
| **Q9** | 0.25 | 0.19 | **0.51** |
| **Q10** | 0.10 | *0.32* | **0.43** |
| **Q11** | 0.07 | 0.16 | **0.59** |
| **Q12** | 0.03 | 0.07 | **0.77** |
| **Q13** | -0.12 | -0.02 | **0.62** |
| **Q14** | -0.05 | -0.06 | **0.88** |
| **Q15** | -0.10 | -0.16 | **0.74** |
| **Q16** | -0.03 | -0.08 | **0.80** |
| **Q17** | -0.00 | 0.02 | **0.82** |

Note: Values in block capitals indicate loadings ≥0.4 employed to determine the final factor structure.

**Table S3: Proportion of Participants in Each Exposure Category According to Quintile of Townsend Deprivation Index**

| **Exposure** | **Quintiles of Townsend Deprivation Index** | | | | | ***p* (χ²)** |
| --- | --- | --- | --- | --- | --- | --- |
| **Neighborhood**  **social cohesion** | **Quintile 1^a^**  **n (%)** | **Quintile 2**  **n (%)** | **Quintile 3**  **n (%)** | **Quintile 4**  **n (%)** | **Quintile 5^b^**  **n (%)** |  |
| *High cohesion* | 784 (27.91) | 319 (23.97) | 353 (24.03) | 305 (19.88) | 110 (17.35) | <.0001 |
| *Medium cohesion* | 1,449 (51.58) | 718 (53.94) | 757 (51.53) | 800 (52.15) | 298 (47.00) |  |
| *Low cohesion* | 576 (20.51) | 294 (22.09) | 359 (24.44) | 429 (27.97) | 226 (35.65) |  |
|  |  |  |  |  |  |  |
| **Neighborhood discord** |  |  |  |  |  |  |
| *Low discord* | 1,857 (66.11) | 793 (59.58) | 865 (58.88) | 854 (55.67) | 301 (47.48) | <.0001 |
| *Medium discord* | 693 (24.67) | 383 (28.78) | 419 (28.52) | 467 (30.44) | 192 (30.28) |  |
| *High discord* | 259 (9.22) | 155 (11.65) | 185 (12.59) | 213 (13.89) | 141 (22.24) |  |
|  |  |  |  |  |  |  |
| **Neighborhood quality** |  |  |  |  |  |  |
| *Low stress* | 2,053 (73.09) | 901 (67.69) | 772 (52.55) | 604 (39.37) | 143 (22.56) | <.0001 |
| *Medium stress* | 714 (25.42) | 397 (29.83) | 599 (40.78) | 751 (48.96) | 311 (49.05) |  |
| *High stress* | 42 (1.50) | 33 (2.48) | 98 (6.67) | 179 (11.67) | 180 (28.39) |  |

**Note:**

^a^ Least deprived.

^b^ Most deprived.

**Table S4: Goodness of Fit/Posterior Probabilities for 2, 3, and 4 Class Solution**

| **Exposure** | **Posterior Probabilities, %** | | | | **Goodness of Fit** | | |
| --- | --- | --- | --- | --- | --- | --- | --- |
| **Social cohesion** | **Trajectory I** | **Trajectory II** | **Trajectory III** | **Trajectory IV** | **AIC** | **BIC** | **2*(BIC rule)** |
| *2-class solution* | 86.252 | 89.598 | **-** | **-** | -192996.51 | -193019.22 | - |
| *3-class solution* | 85.284 | 78.252 | 86.041 | **-** | -190849.32 | -190883.40 | 4271.64 |
| *4-class solution* | 80.719 | 69.983 | 76.415 | 83.378 | -190274.21 | -190319.64 | 1086.0 |
|  |  |  |  |  |  |  |  |
| **Neighborhood discord** |  |  |  |  |  |  |  |
| *2-class solution* | 96.76 | 80.23 | - | - | -46892.57 | -46915.29 | - |
| *3-class solution* | 78.96 | 85.64 | 70.16 | - | -46153.94 | -46188.02 | 1454.54 |
| *4-class solution* | 70.40 | 61.77 | 80.05 | 71.181 | -46028.47 | -46073.91 | 228.22 |
|  |  |  |  |  |  |  |  |
| **Neighborhood quality** |  |  |  |  |  |  |  |
| *2-class solution* | 89.41 | 90.91 | **-** | **-** | -104099.56 | -104121.70 | - |
| *3-class solution* | 90.56 | 83.56 | 77.71 | **-** | -102332.94 | -102366.15 | 3511.1 |
| *4-class solution* | 89.93 | 83.20 | 71.53 | 80.51 | -101646.33 | -101690.61 | 1351.08 |

Note: AIC = Akaike Information Criterion; BIC = Bayesian Information Criterion.

**Table S5: Proportion of Missing Data in Covariates Included in the Analyses**

| **Covariate** | **Proportion of Data Missing (%)** | | | |
| --- | --- | --- | --- | --- |
|  | **Psychotic experiences**  **at 13 years** | **Psychotic experiences**  **at 18 years** | **Depressive symptoms**  **at 13 years** | **Depressive symptoms**  **at 18 years** |
| Gender | 0.04 | 0.02 | 0.05 | 0.00 |
| Ethnicity | 9.80 | 10.44 | 9.81 | 7.38 |
| Maternal age | 7.80 | 8.49 | 7.81 | 6.46 |
| Paternal age | 34.07 | 33.57 | 34.01 | 29.80 |
| Maternal depression | 0.00 | 0.00 | 0.00 | 0.00 |
| Social class | 20.45 | 20.17 | 20.46 | 17.58 |
| Marital status | 7.37 | 7.89 | 7.38 | 5.63 |
| Highest education | 8.46 | 9.05 | 8.46 | 6.51 |
| Area-level deprivation | 44.98 | 45.27 | 44.86 | 44.33 |
| Stressful life event (child) | 34.39 | 34.67 | 34.35 | 28.44 |
| Flu in pregnancy | 12.16 | 13.26 | 12.12 | 10.83 |
| Number of house moves | 42.85 | 42.34 | 42.82 | 36.25 |

**Table S6: Patterns of Missing Outcome Data Across Measures of Exposure and Confounders**

|  | **Psychotic Experiences at Age 13 Years** | | | **Psychotic Experiences at Age 18 Years** | | |
| --- | --- | --- | --- | --- | --- | --- |
|  | **Missing,**  **n (%)** | **Complete,**  **n (%)** | ***p* (χ²)** | **Missing,**  **n (%)** | **Complete,**  **n (%)** | ***p* (χ²)** |
| **Total** | 5,244 (44.8) | 6,455 (55.2) | **-** | 7,273 (62.2) | 4,426 (37.8) |  |
| **Neighborhood cohesion** |  |  |  |  |  |  |
| *High* | 1,142 (21.8) | 1,624 (25.2) | <.0001 | 1,164 (22.2) | 1,152 (26.0) | <.0001 |
| *Medium* | 2,676 (51.0) | 3,359 (52.0) |  | 2,738 (51.4) | 2,297 (51.9) |  |
| *Low* | 1,426 (27.2) | 1,472 (22.8) |  | 1,921 (26.4) | 977 (22.1) |  |
| **Neighborhood discord** |  |  |  |  |  |  |
| *High* | 872 (16.6) | 810 (12.6) | <.0001 | 1,154 (15.9) | 528 (11.9) | <.0001 |
| *Medium* | 1,199 (22.9) | 1,667 (25.8) |  | 1,721 (23.7) | 1,145 (25.9) |  |
| *Low* | 3,173 (60.5) | 3,978 (61.6) |  | 4,398 (60.4) | 2,753 (62.2) |  |
| **Neighborhood stress** |  |  |  |  |  |  |
| *High* | 3,023 (57.7) | 3,703 (57.4) | <.0001 | 4,136 (56.9) | 2,590 (58.5) | <.0001 |
| *Medium* | 1,734 (33.1) | 2,332 (36.1) |  | 2,497 (34.3) | 1,569 (35.5) |  |
| *Low* | 487 (9.3) | 420 (6.5) |  | 640 (8.8) | 267 (6.0) |  |
| **Child’s gender** |  |  |  |  |  |  |
| *Male* | 2,857 (54.5) | 3,172 (49.1) | <.0001 | 4,087 (56.2) | 1,942 (43.9) | <.0001 |
| *Female* | 2,387 (45.5) | 3,283 (50.9) |  | 3,186 (43.8) | 2,484 (56.1) |  |
| **Child’s ethnicity** |  |  |  |  |  |  |
| *White* | 4,431 (95.1) | 5,713 (96.1) | .01 | 6,207 (95.6) | 3,937 (95.9) | .41 |
| *Non-White* | 228 (4.9) | 230 (3.9) |  | 289 (4.5) | 169 (4.1) |  |
| **Child’s experience of SLEs** |  |  |  |  |  |  |
| *No* | 887 (57.4) | 2,509 (57.1) | .82 | 1,169 (%) | 1,727 (%) | .35 |
| *Yes* | 659 (42.6) | 1,889 (42.9) |  | 1,221 (%) | 1,327 (%) |  |
| **Maternal marital status** |  |  |  |  |  |  |
| *Single (i.e., never married)* | 1,037 (21.0) | 827 (13.6) | <.0001 | 1,308 (19.2) | 556 (13.2) | <.0001 |
| *Married* | 3,605 (72.9) | 4,958 (81.6) |  | 5,121 (75.0) | 3,442 (82.0) |  |
| *Widowed/divorced/separated* | 306 (6.1) | 292 (4.8) |  | 398 (5.8) | 200 (4.8) |  |
| **Maternal education** |  |  |  |  |  |  |
| *Vocational* | 1,686 (35.0) | 1,263 (20.9) | <.0001 | 2,191 (32.8) | 758 (18.2) | <.0001 |
| *GCSEs or A Levels* | 2,635 (54.7) | 3,772 (62.5) |  | 3,815 (57.1) | 2,592 (62.2) |  |
| *Degree or higher* | 495 (10.3) | 997 (16.5) |  | 674 (10.1) | 818 (19.6) |  |

|  | **Psychotic Experiences at Age 13 Years** | | | **Psychotic Experiences at Age 18 Years** | | |
| --- | --- | --- | --- | --- | --- | --- |
|  | **Missing,**  **n (%)** | **Complete,**  **n (%)** | ***p* (χ²)** | **Missing,**  **n (%)** | **Complete,**  **n (%)** | ***p* (χ²)** |
| **No. house moves** |  |  |  |  |  |  |
| *Zero/one* | 313 (26.4) | 1,247 (32.3) | <.0001 | 694 (29.7) | 866 (32.0) | <.0001 |
| *Two/three* | 400 (33.8) | 1,456 (37.7) |  | 817 (34.9) | 1,039 (38.3) |  |
| *Four or more* | 471 (39.8) | 1,160 (30.0) |  | 827 (35.4) | 804 (29.7) |  |
| **Maternal depression** |  |  |  |  |  |  |
| *No* | 4,303 (82.1) | 5,533 (85.7) | <.0001 | 6,029 (82.9) | 3,807 (86.0) | <.0001 |
| *Yes* | 941 (17.9) | 922 (14.3) |  | 1,244 (17.1) | 619 (13.9) |  |
| **Flu in pregnancy** |  |  |  |  |  |  |
| *No* | 3,880 (85.4) | 4,957 (86.3) | .17 | 5,431 (85.6) | 3,406 (86.4) | .27 |
| *Yes* | 665 (14.6) | 785 (13.7) |  | 913 (14.4) | 537 (13.6) |  |
| **Maternal social class** |  |  |  |  |  |  |
| *Non-manual* | 2,875 (76.5) | 4,445 (84.6) | <.0001 | 4,198 (78.6) | 3,122 (85.0) | <.0001 |
| *Manual* | 882 (23.5) | 809 (15.4) |  | 1,140 (21.4) | 551 (15.0) |  |
| **Quintiles of area deprivation** |  |  |  |  |  |  |
| *1 (Least deprived)* | 790 (25.4) | 1,060 (29.6) | <.0001 | 1,097 (26.0) | 753 (30.4) | <.0001 |
| *2* | 470 (15.1) | 547 (15.3) |  | 629 (14.9) | 388 (15.7) |  |
| *3* | 573 (18.4) | 671 (18.7) |  | 783 (18.6) | 461 (18.6) |  |
| *4* | 799 (25.7) | 922 (25.7) |  | 1,113 (26.4) | 608 (24.5) |  |
| *5 (Most deprived)* | 482 (15.5) | 385 (10.7) |  | 598 (14.2) | 269 (10.9) |  |
|  | **Missing,**  **Mean (SD)** | **Complete,**  **Mean (SD)** | ***p* (F)** | **Missing,**  **Mean (SD)** | **Complete,**  **Mean (SD)** | ***p* (F)** |
| **Maternal age** | 27.3 (4.9) | 28.8 (4.5) | <.0001 | 27.5 (4.8) | 29.0 (4.6) | <.0001 |
| **Paternal age** | 30.2 (5.8) | 31.3 (5.5) | <.0001 | 30.3 (5.7) | 31,6 (5.5) | <.0001 |

|  | **Depressive Symptoms at Age 13 Years** | | | **Depressive Symptoms at Age 18 Years** | | |
| --- | --- | --- | --- | --- | --- | --- |
|  | **Missing,**  **n (%)** | **Complete,**  **n (%)** | ***p* (χ²)** | **Missing,**  **n (%)** | **Complete,**  **n (%)** | ***p* (χ²)** |
| **Total** | 5,321 (45.5) | 6,378 (54.5) |  | 8,468 (72.4) | 3,231 (27.6) |  |
| **Neighborhood cohesion** |  |  |  |  |  |  |
| *High* | 1,160 (21.8) | 1,606 (25.2) | <.0001 | 1,936 (22.9) | 830 (25.7) | .001 |
| *Medium* | 2,722 (51.2) | 3,313 (51.9) |  | 4,377 (51.7) | 1,658 (51.3) |  |
| *Low* | 1,439 (27.0) | 1,459 (22.9) |  | 2,155 (25.5) | 743 (23.0) |  |
| **Neighborhood discord** |  |  |  |  |  |  |
| *High* | 3,218 (60.5) | 3,933 (61.7) | <.0001 | 1,346 (15.9) | 2,060 (10.4) | <.0001 |
| *Medium* | 1,227 (23.1) | 1,639 (25.7) |  | 2,031 (24.0) | 835 (25.8) |  |
| *Low* | 876 (16.4) | 806 (12.6) |  | 5,091 (60.1) | 336 (63.8) |  |
| **Neighborhood quality** |  |  |  |  |  |  |
| *High* | 495 (9.3) | 412 (12.6) | <.0001 | 4,788 (56.5) | 1,938 (60.0) | <.0001 |
| *Medium* | 1,756 (33.2) | 2,301 (25.7) |  | 2,960 (35.0) | 1,106 (34.2) |  |
| *Low* | 3,061 (57.5) | 3,665 (61.7) |  | 720 (8.5) | 187 (5.8) |  |
| **Child’s gender** |  |  |  |  |  |  |
| *Male* | 2,897 (54.4) | 3,132 (49.1) | <.0001 | 4,867 (57.5) | 1,162 (36.0) | <.0001 |
| *Female* | 2,424 (45.6) | 3,246 (50.9) |  | 3,601 (42.5) | 2,069 (64.0) |  |
| **Child’s ethnicity** |  |  |  |  |  |  |
| *White* | 4,496 (95.1) | 5,648 (96.2) | .006 | 7,229 (95.4) | 2,915 (96.3) | .06 |
| *Non-White* | 233 (4.9) | 225 (50.9) |  | 345 (4.6) | 113 (3.7) |  |
| **Child’s experience of SLEs** |  |  |  |  |  |  |
| *No* | 909 (57.0) | 2,487 (57.2) | .92 | 2,033 (56.8) | 1,363 (57.7) | .51 |
| *Yes* | 685 (43.0) | 1,863 (42.8) |  | 1,547 (43.2) | 1,001 (42.3) |  |
| **Maternal marital status** |  |  |  |  |  |  |
| *Single (i.e., never married)* | 1,055 (21.0) | 809 (13.5) | <.0001 | 1,501 (18.9) | 363 (11.8) | <.0001 |
| *Married* | 3,659 (72.9) | 4,904 (81.7) |  | 5,977 (75.2) | 2,589 (84.0) |  |
| *Widowed/divorced/separated* | 307 (6.1) | 291 (4.9) |  | 468 (5.9) | 130 (4.2) |  |
| **Maternal education** |  |  |  |  |  |  |
| *Vocational* | 1,707 (34.9) | 1,242 (20.8) | <.0001 | 2,430 (31.2) | 519 (17.0) | <.0001 |
| *GCSEs or A Levels* | 2,678 (54.8) | 3,729 (62.6) |  | 4,561 (58.5) | 1,846 (60.4) |  |
| *Degree or higher* | 502 (10.3) | 990 (16.6) |  | 802 (10.3) | 690 (22.6) |  |

|  | **Depressive Symptoms at Age 13 Years** | | | **Depressive Symptoms at Age 18 Years** | | |
| --- | --- | --- | --- | --- | --- | --- |
|  | **Missing,**  **n (%)** | **Complete,**  **n (%)** | ***p* (χ²)** | **Missing,**  **n (%)** | **Complete,**  **n (%)** | ***p* (χ²)** |
| **No. house moves** |  |  |  |  |  |  |
| *Zero/one* | 330 (26.9) | 1,230 (32.2) |  | 885 (30.2) | 675 (31.9) |  |
| *Two/three* | 414 (33.8) | 1,442 (37.7) | <.0001 | 1,074 (36.6) | 782 (37.0) | .22 |
| *Four or more* | 481 (39.3) | 1,150 (30.1) |  | 974 (33.2) | 657 (31.1) |  |
| **Maternal depression** |  |  |  |  |  |  |
| *No* | 4,370 (82.1) | 5,466 (85.7) | <.0001 | 7,037 (83.1) | 2,799 (86.6) | <.0001 |
| *Yes* | 951 (17.9) | 912 (14.3) |  | 1,431 (16.9) | 432 (13.4) |  |
| **Flu in pregnancy** |  |  |  |  |  |  |
| *No* | 3,934 (85.3) | 4,903 (86.4) | .14 | 6,338 (85.8) | 2,499 (86.2) | .58 |
| *Yes* | 676 (14.7) | 774 (13.6) |  | 1,050 (14.2) | 400 (13.8) |  |
| **Maternal social class** |  |  |  |  |  |  |
| *Non-manual* | 2,925 (%) | 4,395 (%) | <.0001 | 4,979 (78.9) | 2,341 (86.8) | <.0001 |
| *Manual* | 894 (%) | 797 (%) |  | 1,334 (21.1) | 357 (13.2) |  |
| **Quintiles of area deprivation** |  |  |  |  |  |  |
| *1 (Least deprived)* | 805 (25.6) | 1,045 (29.4) | <.0001 | 1,307 (26.7) | 543 (30.0) | <.0001 |
| *2* | 477 (15.2) | 540 (15.2) |  | 720 (14.7) | 297 (16.4) |  |
| *3* | 578 (18.4) | 666 (18.7) |  | 886 (18.1) | 358 (19.8) |  |
| *4* | 800 (25.5) | 921 (25.9) |  | 1,300 (26.6) | 421 (23.2) |  |
| *5 (Most deprived)* | 483 (15.4) | 384 (10.8) |  | 675 (13.8) | 192 (10.6) |  |
|  | **Missing,**  **Mean (SD)** | **Complete,**  **Mean (SD)** | ***p* (F)** | **Missing**  **Mean (SD)** | **Complete,**  **Mean (SD)** | ***p* (F)** |
| **Maternal age** | 27.3 (4.9) | 28.8 (4.5) | <.0001 | 27.6 (4.7) | 29.2 (4.5) | <.0001 |
| **Paternal age** | 30.1 (5.8) | 31.3 (5.5) | <.0001 | 30.5 (5.6) | 31.7 (5.6) | <.0001 |

**Note:** GCSE = general certificate of secondary education; SLEs = stressful life events.

**Table S7: Detailed Sample Characteristics in Relation to Psychotic Symptoms at Ages 13/18**

|  | **Psychotic Experiences at Age 13 Years** | | | | **Psychotic Experiences at Age 18 Years** | | | |
| --- | --- | --- | --- | --- | --- | --- | --- | --- |
|  | **n (%)** | **None,**  **n (%)** | **Suspected or definite,**  **n (%)** | ***p* (χ²)** | **n (%)** | **None,**  **n (%)** | **Suspected or definite,**  **n (%)** | ***p* (χ²)** |
| **Total** | 6,455 | 5,719 (88.6) | 736 (11.4) |  | 4,426 | 4,084 (92.3) | 342 (7.7) |  |
| **Child’s gender** |  |  |  |  |  |  |  |  |
| *Male* | 3,172 (%) | 2,835 (49.6) | 337 (45.8) | .053 | 1,942 (43.9) | 1,817 (44.5) | 125 (36.6) | .004 |
| *Female* | 3,282 (%) | 2,884 (50.4) | 399 (54.2) |  | 2,484 (56.1) | 2,267 (55.5) | 217 (63.4) |  |
| **Child’s ethnicity** |  |  |  |  |  |  |  |  |
| *White* | 5,713 (96.1) | 5,067 (96.2) | 646 (95.2) | .69 | 3,937 (95.9) | 3,648 (96.1) | 289 (93.5) | .03 |
| *Non-White* | 230 (3.9) | 202 (3.8) | 28 (4.2) |  | 169 (4.1) | 149 (3.9) | 20 (6.5) |  |
| **Child’s experience of SLEs** |  |  |  |  |  |  |  |  |
| *No* | 2,509 (57.1) | 2,264 (57.7) | 245 (51.4) | .008 | 1,727 (%) | 1,605 (%) | 122 (%) | .56 |
| *Yes* | 1,889 (42.9) | 1,657 (42.3) | 232 (48.6) |  | 1,327 (%) | 1,226 (%) | 101 (%) |  |
| **Maternal marital status** |  |  |  |  |  |  |  |  |
| *Single (i.e., never married)* | 827 (13.6) | 703 (13.0) | 124 (18.1) | <.0001 | 556 (13.2) | 488 (12.6) | 68 (21.4) | <.0001 |
| *Married* | 4,958 (81.6) | 4,448 (82.5) | 510 (74.5) |  | 3,442 (82.0) | 3,218 (82.9) | 224 (70.4) |  |
| *Widowed/Divorced/separated* | 292 (4.8) | 241 (4.5) | 51 (7.5) |  | 200 (4.8) | 174 (4.5) | 26 (8.2) |  |
| **Maternal education** |  |  |  |  |  |  |  |  |
| *Vocational* | 1,263 (20.9) | 1,113 (20.8) | 150 (21.8) | .05 | 758 (18.2) | 675 (17.6) | 83 (26.8) | .001 |
| *GCSEs or A Levels* | 3,772 (62.5) | 3,325 (62.2) | 447 (65.0) |  | 2,592 (62.2) | 2,404 (62.5) | 188 (58.4) |  |
| *Degree or Higher* | 997 (16.6) | 906 (17.0) | 91 (13.2) |  | 818 (19.6) | 767 (19.9) | 51 (15.8) |  |
| **No. house moves** |  |  |  |  |  |  |  |  |
| *Zero/one* | 1,247 (32.3) | 1,123 (32.5) | 124 (30.3) |  | 866 (32.0) | 808 (32.3) | 58 (29.4) |  |
| *Two/three* | 1,456 (37.7) | 1,313 (38.0) | 143 (35.0) | .09 | 1,039 (38.4) | 970 (38.6) | 69 (35.0) | .18 |
| *Four or more* | 1,160 (30.0) | 1,018 (29.5) | 142 (34.7) |  | 804 (29.7) | 734 (29.2) | 70 (35.5) |  |
| **Maternal depression** |  |  |  |  |  |  |  |  |
| *No* | 5,533 (85.7) | 4,933 (86.3) | 600 (81.5) | .001 | 3,807 (86.0) | 3,538 (86.6) | 269 (78.7) | <.0001 |
| *Yes* | 922 (14.3) | 786 (13.7) | 136 (18.5) |  | 619 (14.0) | 546 (13.4) | 73 (21.3) |  |
| **Flu in pregnancy** |  |  |  |  |  |  |  |  |
| *No* | 4,957 (86.3) | 4,421 (86.7) | 536 (83.6) | .034 | 3,406 (86.4) | 3,145 (86.4) | 261 (86.4) | .98 |
| *Yes* | 785 (13.7) | 680 (13.3) | 105 (16.4) |  | 537 (13.6) | 496 (13.6) | 41 (13.6) |  |

|  | **Psychotic Experiences at Age 13 Years** | | | | **Psychotic Experiences at Age 18 Years** | | | |
| --- | --- | --- | --- | --- | --- | --- | --- | --- |
|  | **n (%)** | **None,**  **n (%)** | **Suspected or definite,**  **n (%)** | ***p* (χ²)** | **n (%)** | **None,**  **n (%)** | **Suspected or definite,**  **n (%)** | ***p* (χ²)** |
| **Maternal social class** |  |  |  |  |  |  |  |  |
| *Non-manual* | 4,445 (84.6) | 3,956 (84.8) | 489 (82.7) | .18 | 3,122 (85.0) | 2,906 (85.5) | 216 (78.6) | .002 |
| *Manual* | 809 (15.4) | 707 (15.2) | 102 (17.3) |  | 551 (15.0) | 492 (14.5) | 59 (21.4) |  |
| **Quintiles of area deprivation** |  |  |  |  |  |  |  |  |
| *1 (Least deprived)* | 1,060 (29.6) | 950 (29.8) | 110 (27.8) | .10 | 753 (30.4) | 720 (31.2) | 33 (19.3) | .002 |
| *2* | 547 (15.3) | 481 (15.1) | 66 (16.7) |  | 366 (15.6) | 366 (15.9) | 22 (12.9) |  |
| *3* | 671 (18.7) | 603 (18.9) | 68 (17.2) |  | 461 (18.6) | 423 (18.3) | 38 (22.2) |  |
| *4* | 922 (25.7) | 827 (25.9) | 95 (24.0) |  | 608 (24.5) | 558 (24.2) | 50 (29.2) |  |
| *5 (Most deprived)* | 385 (10.7) | 328 (10.3) | 57 (14.3) |  | 269 (10.9) | 241 (10.4) | 28 (16.4) |  |
|  | **Mean (SD)** | **None,**  **Mean (SD)** | **Suspected or definite,**  **Mean (SD)** | ***p* (F)** | **Mean (SD)** | **None,**  **Mean (SD)** | **Suspected or definite,**  **Mean (SD)** | ***p* (F)** |
| **Maternal age** | 28.8 (4.5) | 28.8 (4.5) | 28.3 (4.7) | .004 | 29.0 (4.6) | 29.1 (4.5) | 27.9 (5.1) | <.0001 |
| **Paternal age** | 31.3 (5.5) | 31.3 (5.5) | 31.2 (5.5) | .55 | 31.6 (5.5) | 31.6 (5.5) | 30.7 (5.3) | .01 |

|  | **Depressive Symptoms at Age 13 Years** | | | | **Depressive Symptoms at Age 18 Years** | | | |
| --- | --- | --- | --- | --- | --- | --- | --- | --- |
|  | **n (%)** | **No,**  **n (%)** | **Suspected depression,**  **n (%)** | ***p* (χ²)** | **n (%)** | **None,**  **n (%)** | **Suspected depression,**  **n (%)** | ***p* (χ²)** |
| **Total** | 6,378 | 6,050 (94.9) | 328 (5.1) |  | 3,231 | 2,639 (81.7) | 592 (18.3) |  |
| **Child’s gender** |  |  |  |  |  |  |  |  |
| *Male* | 3,132 (49.1) | 3,019 (49.9) | 113 (34.5) | <.0001 | 1,162 (36.0) | 1,041 (39.5) | 121 (20.4) | <.0001 |
| *Female* | 3,246 (50.1) | 3,031 (50.15) | 215 (65.6) |  | 2,069 (64.0) | 1,598 (60.5) | 471 (79.6) |  |
| **Child’s ethnicity** |  |  |  |  |  |  |  |  |
| *White* | 5,648 (96.2) | 5,362 (96.1) | 286 (97.3) | .31 | 2,915 (96.3) | 2,389 (96.3) | 526 (96.3) | .93 |
| *Non-White* | 225 (3.8) | 217 (3.9) | 8 (2.7) |  | 113 (3.7) | 93 (3.7) | 20 (3.7) |  |
| **Child’s experience of SLEs** |  |  |  |  |  |  |  |  |
| *No* | 2,487 (57.2) | 2,388 (%) | 99 (%) | .001 | 1,363 (57.7) | 1,125 (58.0) | 238 (56.1) | .48 |
| *Yes* | 1,863 (42.8) | 1,746 (%) | 117 (%) |  | 1,001 (42.3) | 815 (42.0) | 186 (43.9) |  |
| **Maternal marital status** |  |  |  |  |  |  |  |  |
| *Single (i.e., never married)* | 809 (13.4) | 762 (13.4) | 47 (15.4) | .11 | 363 (11.8) | 273 (10.8) | 90 (16.3) | .001 |
| *Married* | 4,904 (81.7) | 4,667 (81.9) | 237 (77.7) |  | 2,586 (84.0) | 2,148 (85.1) | 438 (79.1) |  |
| *Widowed/Divorced/separated* | 291 (4.9) | 270 (4.7) | 21 (6.9) |  | 130 (4.2) | 104 (4.1) | 26 (4.7) |  |
| **Maternal education** |  |  |  |  |  |  |  |  |
| *Vocational* | 1,242 (20.8) | 1,187 (20.9) | 55 (18.2) | .14 | 519 (17.0) | 406 (16.2) | 113 (20.6) | <.0001 |
| *GCSEs or A Levels* | 3,729 (62.6) | 3,524 (62.3) | 205 (67.9) |  | 1,846 (60.4) | 1,497 (59.7) | 349 (63.6) |  |
| *Degree or Higher* | 990 (16.6) | 948 (16.8) | 42 (13.9) |  | 690 (22.6) | 603 (24.1) | 87 (15.9) |  |
| **No. house moves** |  |  |  |  |  |  |  |  |
| *Zero/one* | 1,230 (32.2) | 1,167 (32.1) | 63 (33.7) |  | 675 (31.9) | 552 (31.6) | 123 (33.3) | .60 |
| *Two/three* | 1,442 (37.7) | 1,381 (38.0) | 61 (32.6) | .31 | 782 (37.0) | 654 (37.5) | 128 (34.7) |  |
| *Four or more* | 1,150 (30.1) | 1,087 (29.9) | 63 (33.7) |  | 657 (31.1) | 539 (30.9) | 118 (32.0) |  |
| **Maternal depression** |  |  |  |  |  |  |  |  |
| *No* | 5,466 (85.7) | 5,202 (86.0) | 264 (80.5) | .006 | 2,799 (86.6) | 2,327 (88.2) | 472 (79.7) | <.0001 |
| *Yes* | 912 (14.3) | 848 (14.0) | 64 (19.5) |  | 432 (13.4) | 312 (11.8) | 120 (20.3) |  |
| **Flu in pregnancy** |  |  |  |  |  |  |  |  |
| *No* | 4,903 (86.4) | 4,649 (86.3) | 254 (87.6) | .53 | 2,499 (86.2) | 2,054 (86.1) | 445 (86.6) | .79 |
| *Yes* | 774 (13.6) | 738 (13.7) | 36 (12.4) |  | 400 (13.8) | 331 (13.9) | 69 (13.4) |  |

**Note:** GSCE = general certificate of secondary education; SD = standard deviation; SLEs = stressful life events.

**Table S8: Detailed Sample Characteristics in Relation to Depressive Symptoms at Ages 13/18**

|  | **Depressive Symptoms at Age 13 Years** | | | | **Depressive Symptoms at Age 18 Years** | | | |
| --- | --- | --- | --- | --- | --- | --- | --- | --- |
|  | **n (%)** | **None,**  **n (%)** | **Suspected depression,**  **n (%)** | ***p* (χ²)** | **n (%)** | **None,**  **n (%)** | **Suspected depression,**  **n (%)** | ***p* (χ²)** |
| **Maternal social class** |  |  |  |  |  |  |  |  |
| *Non-manual* | 4,395 (84.6) | 4,162 (84.5) | 233 (87.6) | .17 | 2,341 (86.8) | 1,933 (86.9) | 408 (86.1) | .62 |
| *Manual* | 797 (15.4) | 764 (15.5) | 33 (12.4) |  | 357 (13.2) | 291 (13.1) | 66 (13.9) |  |
| **Quintiles of area deprivation** |  |  |  |  |  |  |  |  |
| *1 (Least deprived)* | 1,045 (29.4) | 993 (29.4) | 52 (28.9) | .87 | 543 (30.0) | 445 (30.3) | 98 (28.5) | .63 |
| *2* | 540 (15.2) | 511 (15.1) | 29 (16.1) |  | 297 (16.4) | 244 (16.6) | 53 (15.4) |  |
| *3* | 666 (18.7) | 636 (18.8) | 30 (16.7) |  | 358 (19.8) | 293 (20.0) | 65 (18.9) |  |
| *4* | 921 (25.9) | 875 (25.9) | 46 (25.6) |  | 421 (23.3) | 336 (22.9) | 85 (24.7) |  |
| *5 (Most deprived)* | 384 (10.8) | 361 (10.7) | 23 (12.8) |  | 192 (10.6) | 149 (10.2) | 43 (12.5) |  |
|  | **Mean (SD)** | **None**  **Mean (SD)** | **Suspected depression,**  **Mean (SD)** | ***p* (F)** | **Mean (SD)** | **None,**  **Mean (SD)** | **Suspected depression,**  **Mean (SD)** | ***p* (F)** |
| **Maternal age** | 28.8 (4.5) | 28.8 (4.5) | 28.7 (4.6) | .64 | 29.2 (4.5) | 29.3 (4.5) | 28.8 (4.7) | .04 |
| **Paternal age** | 31.3 (5.5) | 31.3 (5.5) | 31.2 (5.5) | .75 | 31.7 (5.6) | 31.8 (5.7) | 31.2 (5.4) | .04 |
